# Study of the Role of Void and Residual Silicon Dioxide on the Electrochemical Performance of Silicon Nanoparticles Encapsulated by Graphene

**DOI:** 10.3390/nano11112864

**Published:** 2021-10-27

**Authors:** Dimitrios-Panagiotis Argyropoulos, George Zardalidis, Panagiotis Giotakos, Maria Daletou, Filippos Farmakis

**Affiliations:** 1 Electrical and Computer Engineering Department, Polytechnic School of Xanthi, Democritus University of Thrace, Kimmeria Campus, GR-67100 Xanthi, Greece; gzardali@ee.duth.gr (G.Z.); farmakis@ee.duth.gr (F.F.); 2 Foundation of Research and Technology, Hellas-Institute of Chemical Engineering Sciences, FORTH/ICEHT, Stadiou Str, Platani Rion, P.O. Box 1414, GR-26504 Patras, Greece; pgiotakos@iceht.forth.gr (P.G.); riadal@iceht.forth.gr (M.D.)

**Keywords:** silicon nanoparticles, anode, lithium-ion, graphene, void

## Abstract

Silicon nanoparticles are used to enhance the anode specific capacity for the lithium-ion cell technology. Due to the mechanical deficiencies of silicon during lithiation and delithiation, one of the many strategies that have been proposed consists of enwrapping the silicon nanoparticles with graphene and creating a void area between them so as to accommodate the large volume changes that occur in the silicon nanoparticle. This work aims to investigate the electrochemical performance and the associated kinetics of the hollow outer shell nanoparticles. To this end, we prepared hollow outer shell silicon nanoparticles (nps) enwrapped with graphene by using thermally grown silicon dioxide as a sacrificial layer, ball milling to enwrap silicon particles with graphene and hydro fluorine (HF) to etch the sacrificial SiO_2_ layer. In addition, in order to offer a wider vision on the electrochemical behavior of the hollow outer shell Si nps, we also prepared all the possible in-between process stages of nps and corresponding electrodes (i.e., bare Si nps, bare Si nps enwrapped with graphene, Si/SiO_2_ nps and Si/SiO_2_ nps enwrapped with graphene). The morphology of all particles revealed the existence of graphene encapsulation, void, and a residual layer of silicon dioxide depending on the process of each nanoparticle. Corresponding electrodes were prepared and studied in half cell configurations by means of galvanostatic cycling, cyclic voltammetry and electrochemical impedance spectroscopy. It was observed that nanoparticles encapsulated with graphene demonstrated high specific capacity but limited cycle life. In contrast, nanoparticles with void and/or SiO_2_ were able to deliver improved cycle life. It is suggested that the existence of the void and/or residual SiO_2_ layer limits the formation of rich Li_X_Si alloys in the core silicon nanoparticle, providing higher mechanical stability during the lithiation and delithiation processes.

## 1. Introduction

Lithium-ion Batteries (LIBs) are currently the most prominent choice for energy storage applications, especially for portable electronic devices, cordless power tools and electric vehicles thanks to their high energy density, long cycle life and low weight, compared to other energy storage technologies [1,2]. However, there is a continuous need to improve the overall electrochemical performance of lithium-ion batteries, as the constant technological advances in the fields of automotive industry and portable communication device industry demand higher specific energy and energy density (>400 Wh/kg and >800 Wh/L) [3] than actually commercially available cells.

Commonly, LIBs use graphite-based anodes (negative electrode), which have a capacity limitation due to the theoretical specific capacity of graphite versus Li/Li^+^, ~372 mAh/g, with a lithiation phase of LiC_6_ [4]. The most promising material to replace graphite is silicon (Si) with a maximum specific capacity, at room temperature, of 3579 mAh/g at the lithiation phase of Li_15_Si_4_ [5,6], offering, in addition, a low potential vs Li/Li^+^, abundance, non-toxicity and huge know-how processing from the microelectronic industry. However, pure silicon anodes are not ready to be commercialized, as silicon expands during lithiation (up to 300% of its initial volume) resulting in electrode cracking, material pulverization and Solid Electrolyte Interphase (SEI) fracture and regrowth [4,7,8]. Consequently, pure silicon electrodes demonstrate a continuous capacity fading upon cycling leading to limited cell cycle life.

During the past years, there has been extensive ongoing research on improving the cycle life of silicon electrodes. Different approaches have been adopted to address the expanding problem of silicon, by focusing on different aspects of the electrode. One approach has been the investigation of different binding materials, targeting to enhance the mechanical stability of silicon electrode by increasing its elasticity. Binding materials, such as polyacrylic acid (PAA) [9,10,11,12,13,14], carboxymethyl cellulose (CMC) [15,16,17], styrene butadiene rubber (SBR) [18,19], and combinations of those binders [18,19,20,21,22] have been widely used. Namely, Zeng et al. [23] proposed a polymer binder of cross-linked polyethylene oxide (PEO), polyethylenimine (PEI) and 3,4-ethylenedioxythiopene poly(styrenesulfonate) (PEDOT:PSS), offering high Li-diffusivity, electron conductivity and yield high modulus, which resulted in a specific capacity of 2027 mAh/g after 500 cycles.

An alternative approach has been the preparation and investigation of Si nanostructures [24,25,26,27,28,29,30] that can accommodate the volume expansions during cycling, by manipulating the size, the surface and the morphology of silicon particles. Liu et al. [29] found that below the critical silicon particle size of ~150 nm cracking and fracturing did not occur during lithiation and delithiation. Furthermore, in order to enhance the electrical conductivity of silicon and offer a protective layer for SEI formation, the use of carbon-based coatings [31,32,33,34,35,36,37,38] has been proposed, preventing the continuous cracking and growth of SEI. Towards this direction, Mu et al. [39] reported electrodes composed of silicon nanoparticles stabilized in a robust graphite-carbon architecture that delivered 620 mAh/g after 100 cycles at high mass loadings. Zhang et al. [40] proposed an electrode with stacking-like coverage of silicon with graphene that was able to cycle for over 1000 cycles at ~1200 mAh/g, and a first coulombic efficiency of ~58%.

Finally, a promising approach focuses on combining two of the above-mentioned strategies, i.e., the creation of silicon nanostructures and the use of a carbon-based shell/coating. In addition, by managing to create a void space in between silicon and the carbon shell [38,41,42,43,44,45,46,47] the nanoparticle can swell during lithiation without fracturing the outer shell and the corresponding SEI that is formed on the carbon shell surface. Therefore, silicon nanoparticles could accommodate the large expansion, thus leading to enhanced mechanical stability and increased electrical conductivity. Initially, Chen et al. [46] reported a silicon core-hollow (void) carbon shell which exhibited better cycle life than bare silicon nanoparticles with first coulombic efficiency of ~65% and 1100 mAh/g after 300 cycles. Liu et al. [43] proposed a similar structure, noted as “yolk-shell”, that provided more than 800 cycles with a retained capacity of 1500 mAh/g. Shuri et al. [48] investigated the synthesis and electrochemical performance of silicon hollow and porous spheres coated with carbon via carbonization of a pyrrole precursor, delivering ~1000 mAh/g after 120 cycles at 0.5 A/g with a capacity retention of ~45%. Hou et al. [49] fabricated a silicon void carbon nanohybrid, by SiO_2_ creation, Chemical Vapor Deposition (CVD) deposited carbon shell and HF etching. Even though many groups have proposed similar structures with successful results on galvanostatic cycling, it remains unclear whether the void simply acts as an available space to the silicon swelling or also is implicated to the lithium kinetics during lithiation and delithiation. In addition, the role of the remaining SiO_2_ on top of the silicon nanoparticle is a factor that has not yet been highlighted, which is accentuated by the various applied oxidation techniques.

The scope of this work is to investigate the electrochemical performance and the associated kinetics of silicon nanoparticles encapsulated by a graphene shell with a void in the outer area of the silicon core. In order to investigate the role of the void and the remaining SiO_2_ grown by a standard dry thermal oxidation, we prepared all possible in-between process stages, related nanoparticles and corresponding electrodes, and performed morphological and electrochemical characterization.

## 2. Materials and Methods

### 2.1. Materials

Silicon nanoparticles (Si nps, 99%, average diameter 100 nm) were purchased from SkySpring Nanomaterials, Inc. (Houston, TX, USA). Graphene nanoplatelets (750 m^2^/g) were obtained from Sigma-Aldrich Biochemical Co., Ltd. (Merck KGaA, Darmstadt, Germany). Hydrofluoric Acid (40%) was purchased from Sigma-Aldrich Biochemical Co., Ltd. (Merck KGaA, Darmstadt, Germany). Super Conductive Carbon Black Powder (Super P CB) and Carboxymethyl Cellulose (CMC) were acquired from Xiamen Tmax Battery Equipments Limited (Xiamen, Fujian, China).

### 2.2. Synthesis of Hollow Outer Shell Si Nanoparticles

Si nanoparticles with an average diameter of 100 nm were thermally oxidized at 900 °C for 1 h to create an oxide outer layer with a thickness of 14 nm, according to the duration and temperature of the oxidation process. The prepared Si/SiO_2_ powders were wet ball milled with graphene for 30 min at 500 rpm, to create around the Si/SiO_2_ particles a graphene shell, that consists of graphene platelets interconnected with each other due to the ball milling process. For removing the SiO_2_ by chemical etching, and thus create a void area, the resulting mixture (Si/SiO_2__Gr) was exposed to an 8% HF solution in Deionized (DI) water for 1 min, centrifuged at 4000 rpm for 15 min and thoroughly washed with DI water multiple times and dried in an ambient environment at 50 °C for a day. The resulting powder was noted as Si/void_Gr. It has to be noted that the chemical etching duration is very important for the successful creation of the graphene shell; it was found that for extensive chemical etching process, the interconnection between graphene nanoplatelets was lost and some nanoplatelets might have been removed, resulting in the exposure of the silicon core.

For comparison reasons, silicon enwrapped with graphene platelets (noted as Si_Gr) was fabricated by using the same ball milling process as described above.

### 2.3. Physical Characterization

Transmission electron microscopy (TEM) and high resolution (HR) TEM were performed on a JEOL JEM2100 operating (JEOL Ltd., Akishima, Tokyo, Japan) at 200 kV. For the preparation of the samples, carbon coated copper grids were used.

### 2.4. Electrode and Cell Preparation

**Figure 1:**
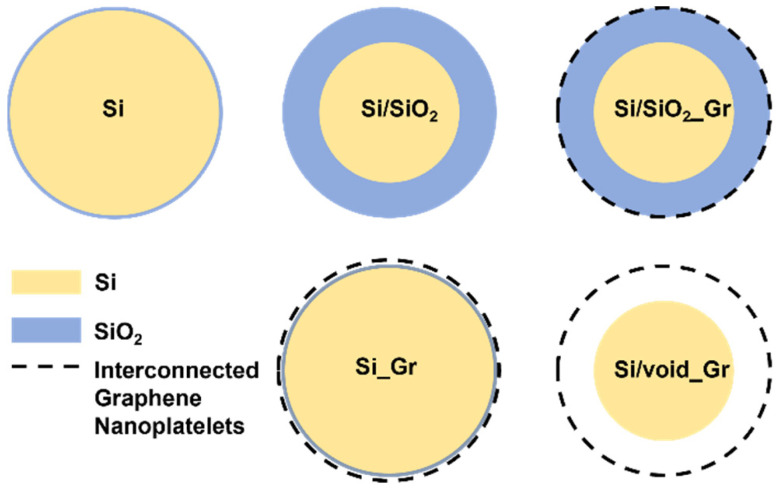
Graphical representation of various prepared nanoparticles in the corresponding electrodes. Dimensions are not in scale.

For the electrochemical characterization five different electrodes were prepared using the same slurry mixing process. The active material (AM) was wet ball milled, in deionized water using a planetary ball milling equipment, along with Super P Carbon Black for 30 min at 500 rpm, then carboxymethyl cellulose (CMC) was added and mixed for 2 h with a custom-made overhead mixer at 400 rpm. The ratio of the slurries AM/SuperP/CMC was 6/2/2. All slurry mixtures were pasted onto copper (Cu) foil with the aid of doctor’s blade film applicator (Xiamen Tmax Battery Equipments Limited, Xiamen, Fujian, China), applying a gap of 10 μm and then dried at 40 °C for a day. The resulting electrodes were rolled, pressed at a calendering machine (Xiamen Tmax Battery Equipments Limited, Xiamen, Fujian, China), and cut into disks of 1.131 cm^2^ of surface. The mass loading of the active material for all electrodes was between 0.45 to 0.6 mg/cm^2^. Table 1 summarizes the composition information for all five fabricated electrodes and Figure 1 illustrates a graphical representation of all prepared particles that are implemented in the electrodes. In order to characterize the electrodes, half cells were assembled in an argon filled glove box, with Li metal as counter electrode and 150 μL LP30 + 2% Fluoroethylene carbonate (FEC) as electrolyte. For obtaining additional information on the electrochemical performance and exclude cell parameters such as internal cell pressure, amount of the electrolyte and separator, two types of test half-cells were prepared: CR2032 coin-type for all electrodes, with Celgard 2500 as separator, and Swagelok^®^ T-type half-cell for the electrodes Si_Gr and Si/void_Gr, with Scimat MG38115 as separator.

### 2.5. Electrochemical Characterization

Galvanostatic charge-discharge tests were performed using BaSyTec CTS (BaSyTec GmbH, Asselfingen, Baden-Württemberg, Germany) from 20 mV to 1.2 V. For the electrochemical impedance spectroscopy (EIS) measurements, PalmSens3 Impedance Analyzer (Palmsens BV, Houten, The Netherlands) was used within a frequency window of 50 kHz to 0.01 Hz. Cyclic Voltammetry (CV) was conducted with PalmSens EmStat3 potentiostat (Palmsens BV, Houten, The Netherlands) from 1.5 V to 10 mV using six scan rates of 0.1 mV/s, 0.2 mV/s, 0.5 mV/s, 0.8 mV/s, 5 mV/s, and 8 mV/s. For the calculation of the specific capacity (i.e., mAh/g), the active mass (silicon nps and graphene) was taken into consideration.

## 3. Results & Discussion

### 3.1. Physical Characterization

**Figure 2:**
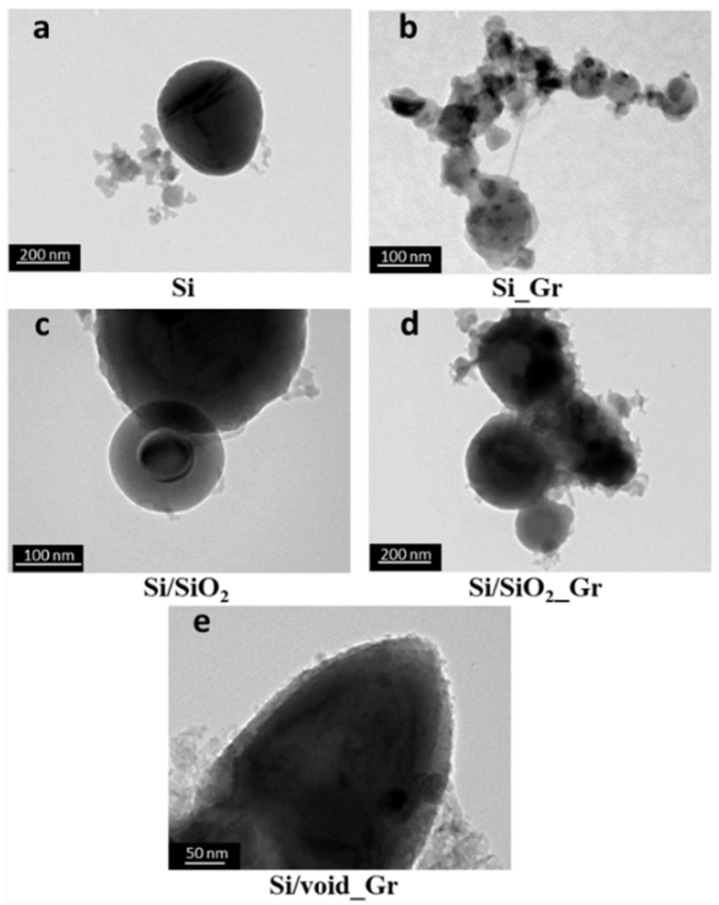
TEM images of the different silica nanoparticles-based samples listed in Table 1, of (**a**) Si, (**b**) Si _Gr, (**c**) Si/SiO_2__Gr, (**d**) Si/SiO_2__Gr, and (**e**) Si/void_Gr.

**Figure 3:**
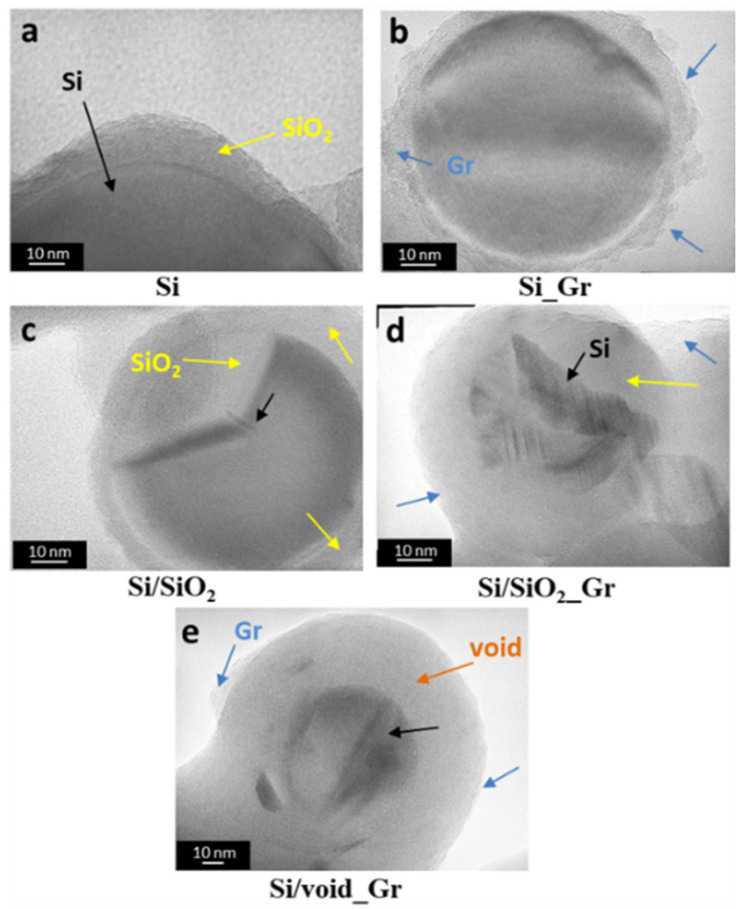
High Resolution TEM images of selected samples listed in Table 1, of (**a**) Si, (**b**) Si_Gr, (**c**) Si/SiO_2_, (**d**) Si/SiO_2__Gr, and (**e**) Si/void_Gr powders. Black arrows: silicon, yellow arrows: SiO_2_, blue arrows: graphene layer, and orange arrow: void.

Figure 2 and Figure 3 show TEM and HR-TEM images, respectively, of the different samples that are listed in Table 1. The Si commercial sample consists mainly of spherical silicon nanoparticles with a broad size distribution and diameters ranging between 50 to 200 nm (Figure 2a). As expected, the corresponding HR-TEM of the same sample in Figure 3a revealed a small layer of oxide (yellow arrows) around 5–10 nm that is surrounding the main Si crystalline core (black arrows). After oxidation of the initial Si sample resulting in Si/SiO_2_, the oxide layer increased (Figure 2c and Figure 3c), thus decreasing the Si core, without any other significant change in the overall sample morphology. On the other hand, changes were observed after the addition of graphene. In the Si_Gr sample (Figure 2b and Figure 3b), the majority of the nanoparticles were surrounded by the graphene layers while the same is also observed for the Si/SiO_2__Gr sample, as shown in Figure 2d and Figure 3d. It seems that no agglomeration took place, but many individual Si nanoparticles were enwrapped with the same graphene structure forming an interconnected network of graphene nanosheets [50]. In Figure 3, for both Si_Gr (Figure 3b) and Si/SiO_2__Gr (Figure 3d) samples, the blue arrows point to the outer layer where the lamellar-structured graphene is observable. Moreover, for the Si/SiO_2__Gr sample, the Si crystalline core can be observed as the darker areas in the center of the less dense oxide layer (yellow arrow) with a thickness of about 15–20 nm.

A subsequent treatment of Si/SiO_2__Gr with the HF solution (etching) was performed in order to remove part of the SiO_2_, applying an etching time of 1 min, resulting in the Si/void_Gr sample. As can be seen in Figure 2e, the HF treatment followed for the Si/void_Gr sample slightly affected but did not remove the graphene from the surface of the Si nanoparticles. These observations coincide with the HR-TEM image shown in Figure 3e, where the Si/void_Gr sample presents three distinct areas, namely the Si core, the SiO_2_-void area (orange arrow) formed after the partial removal of the oxide and the graphene outer layer. The extent and composition of the SiO_2_-void area depends on parameters like oxidation time (thickness of SiO_2_) and the etching time. In literature, Si cores of 50 nm have been reported with definite void areas from the complete removal of the oxide, contact points with the outer carbon shell and void thicknesses ranging from several tens of nm [44], up to 80–100 nm [49]. In the case of the Si/void_Gr sample of this work, a smaller almost centered core is surrounded by a mixed (SiO_2_ and void) porous layer with a thickness of 10–30 nm.

### 3.2. Electrochemical Characterization

**Figure 4:**
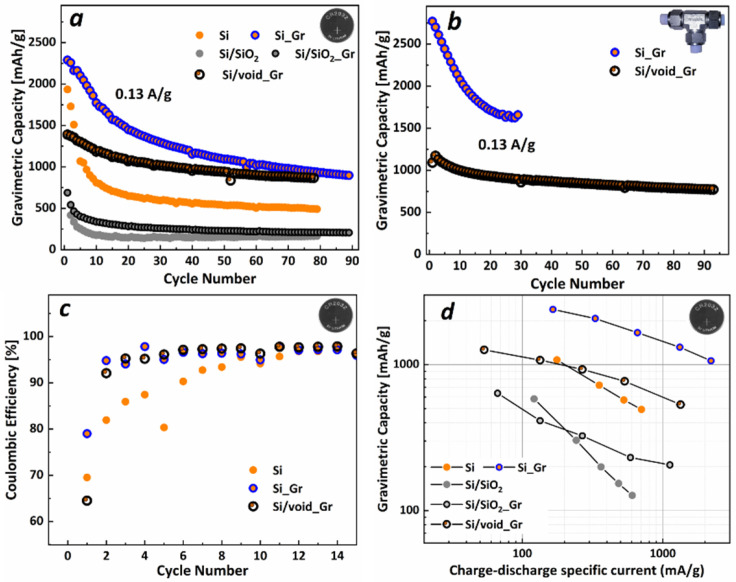
(**a**,**b**) Discharge gravimetric specific capacity in (**a**) CR2032 coin-type half-cells and (**b**) Swagelok^®^ T-type half-cells. (**c**) Coulombic efficiency for electrodes Si, Si_Gr, and Si/void_Gr in coin-type half-cells and (**d**) Gravimetric specific capacity as a function of the charge-discharge specific current for all electrodes.

Figure 4a–c present the cycling performance of all mentioned electrodes, in CR2032 coin-type half-cell and in Swagelok^®^ T-type half-cell configuration, respectively. Si electrodes exhibit 1900 mAh/g specific capacity at the 1st cycle with 69.5% Coulombic efficiency (CE) and loses 20% of its initial capacity at the 3rd cycle and the 50% after 10 cycles. From the 25th cycle and up to 80 cycles the capacity was maintained at ~550 mAh/g. For the Si nanoparticles that were enwrapped with graphene (Si_Gr), the first discharge capacity was 2386 mAh/g with CE of 81%, higher than the bare Si nps, which is another demonstration of the role of graphene to the kinetics of the Si nps. However, the Si_Gr electrode degraded quickly, reaching 80% of its initial capacity after 8 cycles and 50% at cycle 18, showing that the silicon and SEI fracturing during lithiation could not be prevented by the graphene cover. Si/SiO_2_ electrode exhibited, as expected, an initial low capacity of 638 mAh/g with 66% CE, reached 80% of initial capacity after 3 cycles and 50% after the following 3 cycles. Si/SiO_2__Gr returned 688 mAh/g with 60% CE after the 1st lithiation, lost 20% of initial capacity in the 2nd cycle and then another 30%, reaching 50% of initial capacity after 10 cycles. Finally, the electrode with Si/void_Gr exhibited a moderate initial specific capacity of 1390 mAh/g with 64.5% CE. However, for this electrode, 80% of initial capacity was reached after 18 cycles, and maintained a specific capacity of 867 mAh/g after 78 cycles. The voltage profiles of the above electrochemical results can be observed in Figure S1. The electrochemical performance is comparable with previous reported works [41,42,43,44,46,47,48,49], as can be seen in Table 2. From Figure 4b, we can observe that the cycling performance in a Swagelok^®^ T-type half-cell is similar to that of a coin-type half-cell, thus the pressure, the amount of electrolyte and the type of the separator have not significantly affected cell performance.

Figure 4c shows the CE for the electrodes Si, Si_Gr, and Si/void_Gr from the coin-type cells configuration tests. The 1st CE for Si was found to be 70% and for Si/void_Gr it was 65%, whereas for Si _Gr the 1st CE was 80%. For the next 6 cycles, CE of Si increased from 80 to 90%, finally reaching over 98% after 12 cycles and remaining there for the rest of the cycles. On the other hand, during the next 6 cycles, CE for Si_Gr and Si/void_Gr increased from 92% to 96%, reaching 98% after 12 cycles and stabilizing after 20 cycles at 99%. By comparing CE from Si and Si_Gr electrodes, it can be suggested that graphene affects the creation of the SEI by enhancing electrode ion kinetics.

As far as the current rate capability of the electrodes is concerned, Figure 4d illustrates the gravimetric specific capacity as a function of the charge/discharge specific current for all prepared electrodes. It can be observed that, on one hand, for all electrodes having graphene coated nanoparticles, the decrease of the capacity for higher current rates is similar; however, with different capacity values. On the other hand, Si and Si/SiO_2_ electrodes exhibit a more pronounced capacity drop for increasing the charge-discharge current, which can be attributed to their lower kinetics. Regarding the electrode containing Si/void_Gr nanoparticles, it showed a capacity loss of around 50 mAh/g for every doubling of the charge/discharge current value.

In order to further investigate the lithiation and delithiation mechanisms of the various electrodes, cyclic voltammetry is a common method and the peaks that appear in the voltammograms are related to the possible phase transformations and the redox reactions that occur within the electrodes. To this purpose, CV has been performed at 0.1 mV/s for the first 5 cycles and at 0.2 mV/s, 0.5 mV/s, 0.8 mV/s, 5 mV/s and 8 mV/s scan rates for the 6th to 10th cycles, respectively.

**Figure 5:**
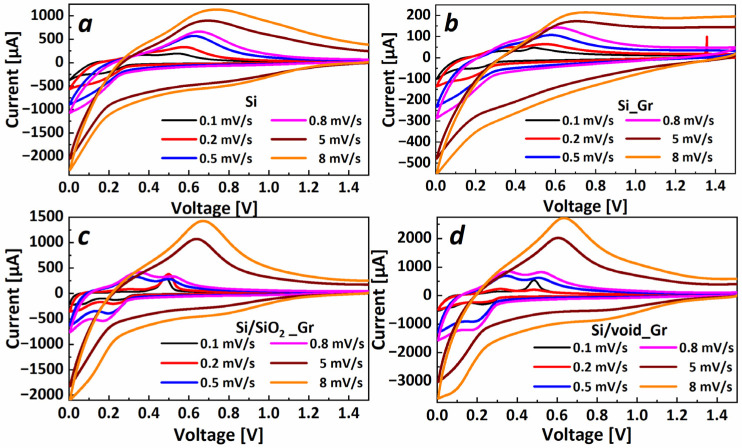
Cyclic Voltammetry from 5th to 10th cycles at 0.1 mV/s, 0.2 mV/s, 0.5 mV/s, 0.8 mV/s, 5 mV/s, and 8 mV/s scan rates, respectively, for (**a**) Si nps, (**b**) Si_Gr, (**c**) Si/SiO_2__Gr, and (**d**) Si/void_Gr.

Figure 5 shows the cyclic voltammograms for the electrodes Si, Si_Gr, Si/SiO_2__Gr, and Si/void_Gr. The cell with Si electrode, at the slowest scan rate, exhibits two cathodic peaks (Figure 5a), the first at ~200 mV corresponding to the lithiation phase of the amorphous Li_7_Si_3_ to the amorphous Li_3.16_Si, and the second peak at the end of lithiation, in our case 5 mV, where the lithiation phase is shifting from the amorphous Li_3.16_Si to crystalline Li_4.25_Si [51]. At the delithiation (anodic branch), we can observe the two equivalent peaks, one at ~375 mV which corresponds to the phase transition from amorphous Li_3.16_Si to amorphous Li_2.3_Si and the second broad anodic peak at ~540 mV which corresponds to amorphous Li_2.3_Si to amorphous Si. With increasing scan rate, the two cathodic peaks appear less distinctive and the anodic peak at 300 mV seems to be suppressed compared to the peak at 540 mV. Especially for the second anodic peak (at 540 mV in the case of 0.1 mV/s), it is observed that with increasing scan rate, the peak shifts toward positive potential values; for 0.8 mV/s rate, as compared to 0.1 mV/s, the peak has shifted more than 100 mV due to electrode kinetics [52]. Similar behavior can be seen for the Si_Gr electrode, in Figure 5b, with the difference to the Si sample, that all corresponding peaks appear at lower potentials of around 50 mV, which is mostly due to the higher electron conduction within the electrode provided by the addition of graphene. This is also supported by the higher discharge capacity of the Si_Gr electrode compared to the Si electrode during the first galvanostatic cycle (Figure 4a). Finally, in the case of high scan rates, a high cathodic current within a potential range between 0.6 V and 1 V is an indicator of electrolyte decomposition and SEI generation. From the cycling performance of those electrodes, the large capacity fading versus cycling is demonstrated.

Regarding the Si/SiO_2__Gr electrode (Figure 5c), four cathodic peaks can be observed at the scan rate of 0.1 mV/s at ~270 mV, ~226 mV, ~63 mV and 24 mV, respectively. These peaks are attributed to the lithiation phases of Si nps, that is, in the core of the particle. For moderate scan rates (up to 0.8 mV/s), it can be observed that only two anodic peaks are distinctly visible that correspond to the lithiation phases of Si that slightly shift toward more negative potential values for the increasing scan rate. For the anodic curve, at 0.1 mV/s, two peaks are visible: the low current broad peak at ~300 mV (P1) and a high current narrow peak at 500 mV (P2). For moderate scan rates up to 0.8 mV/s, the peak at ~300 mV, corresponding to the delithiation phase transitions of rich Li_x_Si alloys to moderate ones, becomes more pronounced while the peak at 500 mV slightly increases and shifts to positive values. In the case of high scan rates of 5 mV/s and 8 mV/s, the curves resemble the Si and Si_Gr electrodes. In the case of the Si/void_Gr electrode, the voltammograms (Figure 5d) look similar to that of the Si/SiO_2__Gr electrode (Figure 5c), with similar peak intensity, position and evolution for a higher scan rate. 

**Figure 6:**
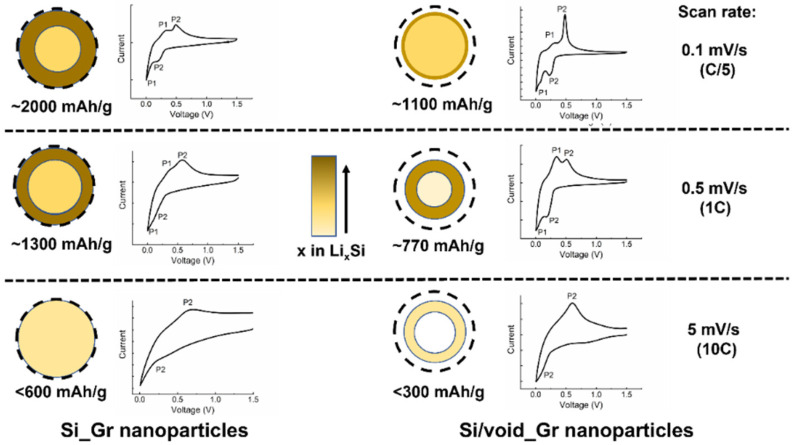
Schematic representation of the lithiated state at 0 V for Si_Gr and Si/void_Gr nanoparticles at various scan rates (and related C rates) with the corresponding voltammograms. The thickness of the various alloys, nanoparticles and voids is not to be considered as accurately defined.

Therefore, we observe that the difference in the evolution of peaks at the various scan rates between the Si (and Si_Gr) electrodes and the electrodes containing either SiO_2_ or void as outer layers, is based on the kinetics of the electrodes and the related transport mechanisms. Indeed, the Si/SiO_2__Gr and Si/void_Gr electrodes present a lower electron conduction, leading to a higher polarization and eventually lower specific capacity as supported by Figure 4. This said, x, in Li_x_Si alloys, takes lower values for those electrodes compared to Si and Si_Gr electrodes at 0.1 mV/s and this is the reason why no delithiation peak (P1) is pronounced at low scan rates. For moderate scan rates, the delithiation P1 peak is more distinctive, for electrodes Si/SiO_2__Gr and Si/void_Gr, due to the low kinetics and the inability of lithium to effectively diffuse inside the core of the nanoparticle. Thus, a rich Li_x_Si alloy is formed at the surface, whereas a lower amount of lithium accesses the center of the nanoparticle. Finally, for high scan rates, only a moderately rich alloy is created within a region around the nanoparticle. Figure 6 graphically suggests a possible scenario of the lithiated states for the Si_Gr and the Si/void_Gr nanoparticles along with the capacity values when the electrodes were cycled at various C-rates and the associated CV curves. It has to be noted that this graphical approach serves as a guide to illustrate the lithiation and delithiation mechanisms of the different nanoparticles.

**Figure 7:**
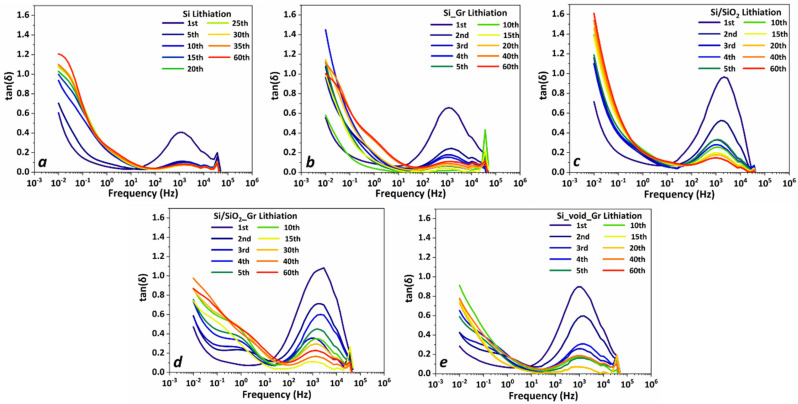
Loss tangent spectra, at the end of each lithiation cycle, for lithium half cells with cathodes of (**a**) Si, (**b**) Si_Gr, (**c**) Si/SiO_2_, (**d**) Si/SiO_2__Gr, and (**e**) Si/void_Gr.

In order to further investigate the SEI characteristics, electrochemical impedance spectroscopy (EIS) analysis was carried out in all electrodes. To determine the ion transport dynamic characteristics in the various local environments developed as electrode electrolyte interphases, the loss tangent spectra are analyzed. Figure 7 shows the loss tangent of half cells with Si, Si/SiO_2_, Si_Gr, Si/SiO_2__Gr and Si/void_Gr electrodes from their impedance measurements taken at the end of each lithiation cycle. All tan(δ) spectra show a prominent peak at high frequencies around 1 kHz, that is decreased in intensity after the first cycle. A second peak can be distinguished in the mid frequency range, between 0.1 Hz and 10 Hz, for all the systems that contain graphene: Si_Gr, Si/SiO_2__Gr and the Si/void_Gr in Figure 7b,d,e. At low frequencies, below 0.1 Hz the characteristic feature of finite space Warburg (FSW) diffusion is evident, as a steep increase in tan(δ) value.

**Figure 8:**
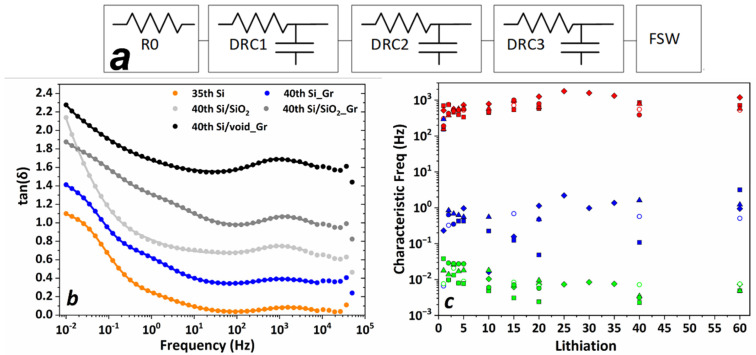
(**a**) Electrical response model for the description of the tan(δ) data composed by a resistance in series with three distributed RC elements and a finite space Warburg element. (**b**) representative fitted curves (continuous lines) for the impedance data after the 40th lithiation. (**c**) Characteristic frequencies of the three peaks from tan(δ) spectra for Si (rhombi), Si_Gr (open circles), Si/SiO_2_ (squares), Si/SiO_2__Gr (triangles), and Si/void_Gr (filled circles).

Based on these observations, the impedance behavior for all the systems has been modeled using the electrical response model shown in Figure 8a comprising an in-series resistance R0 modeling the bulk electrolyte resistance, three distributed RC (*DRC*) equivalent circuits [53], to account for the different environments through which lithium ions move on their way from the anode to the cathode and the FSW element for the diffusion of lithium in the active material. The *DRC* model constitutes an extension of the empirical Havriliak–Negami function [54] where a distribution of characteristic ion transport times is expressed as a broadening of impedance spectral features. The *DRC* impedance is given by the equation

(1) $$Z_{DRC}=\frac{R}{{\left(1+{\left(i\omega \tau \right)}^{1-a}\right)}^{b}}$$

where *τ* = *RC* is the characteristic relaxation time of ion hopping, *R* is the parallel resistance, C is the equivalent capacitance, and *a* and *b* are the fractional shape parameters with 0 < *a* ≤ 1 and 0 < *b* ≤ 1. In the special case of *a* = 0 and *b* = 1 the *RC* parallel equivalent circuit is modeled, while *b* = 1 and *a* < 1 models the parallel resistance–Constant Phase Element (CPE) circuit with a symmetric frequency distribution broadening centered at *τ*. In general, when *b* < 1, an asymmetric broadening appears on the high frequency wing of the impedance spectrum.

The *FSW* element impedance is associated with diffusion where one of the boundaries is blocking, as it is the case in porous electrodes and is given by

(2) $$Z_{FSW}=Z_{0}{\left(i\omega \tau \right)}^{-\frac{1}{2}}coth\;coth\;{\left(i\omega \tau \right)}^{\frac{1}{2}}\;$$

where *Z*_0_ is the Warburg coefficient and *τ* parametrizes a characteristic time where the low frequency capacitive-like behavior emerges.

All tan(δ) curves were fitted using this model and representative fits for all systems after the 40th lithiation are shown in Figure 8b.

The analysis of the fitted curves revealed the existence of three peaks that evolve in three distinct spectral regions, as shown in Figure 8c. The position, spectral range, and consistent time evolution of the high frequency peak around 1 kHz throughout all systems indicate that it must be related to the interphase (SEI) formed on the lithium electrode. The other two peaks are differentiated in each system, however there are some common features. The peak appearing in the mid-frequency range seems to be more pronounced in graphene-containing systems, hence it could be related to components developed on the graphene layer encapsulating the cores of Si or Si/SiO_2_. It should be pointed out that spectra of non-graphene bearing Si and Si/SiO_2_ electrodes also contain a peak in this region but with significantly lower intensity. The existence of common chemical species that form the interphase of both graphene and non-graphene containing systems, such as Super P carbon black and carboxymethyl cellulose could explain this behavior, however, it appears to a higher concentration in the graphene-containing ones, thus we believe it is related to components containing carbon.

The existence of a third low frequency peak, below 0.1 Hz, is evident by the distortion of the lithium diffusion feature, which takes place in the same spectral region. This distortion can be clearly seen for example in Si_Gr 40th and the 60th lithiation cycle of Figure 7b. This is a common feature to all the studied systems, and it is attributed to surface layers formed on Si and SiO_2_ [55]. From the evolution of the systems, as monitored by impedance spectroscopy it can be stated that the ion dynamics at the interphases are very similar; however, it must be pointed out that this picture represents the state of the systems at the end of each lithiation cycle.

Table 3 shows the Warburg coefficient values for all electrodes for the first five and 20 and 40 galvanostatic cycles. As it is generally accepted, the Warburg element is directly related to the diffusion processes of Li atoms to the active material, in our case the silicon nanoparticle. It can be observed that Si_Gr electrode demonstrates the highest diffusion coefficient in the first and the following four cycles which is in agreement with our previous analysis. On the other hand, Si/void_Gr exhibits a rather high diffusion coefficient in the first cycle that drastically deteriorates over the next four cycles, even though its capacity remains stable for a large number of cycles, as shown in Figure 4. It is interesting to note that the Si/void_Gr and Si/SiO_2__Gr system share similar Warburg coefficients after the 1st galvanostatic cycle, and it merits further investigation to identify the roles of SiO_2_ and the void in the diffusion of lithium inside the silicon.

Therefore, in addition to the mechanism proposed by previous studies (Table 2), i.e., the accommodation of volume expansion during a lithiation/delithiation cycle, we suggest that void and SiO_2_ contribute as limiting factors to the lithiation level of the silicon nanoparticle.

## 4. Conclusions

Nanostructured silicon nanoparticles encapsulated by graphene with a void space in between the Si core and the graphene shell were fabricated, using silicon dioxide as a sacrificial layer, which was removed with an HF chemical etching treatment. The physical characterization via TEM and HRTEM demonstrated the evolution in the morphology of the active material, along the process stages from silicon nanoparticle to the final void containing nanostructures. The void-area containing nanoparticles (Si/void_Gr) exhibited a morphology of a Si core with a residual SiO_2_, a void space ranging from 10–20 nm and a graphene outer shell. The electrochemical analysis supported the morphological analysis, through galvanostatic cycling, CV and EIS, as the Si/void_Gr electrode delivered a stable performance upon cycling with a specific capacity, referring to Swagelok^®^ type half-cell configuration, of ~1090 mAh/g at the first cycle and ~798 mAh/g after 75 cycles. The analysis of the various Si and Si/SiO_2_ (with and without graphene encapsulation) nanoparticles revealed that SiO_2_ and/or void area results in more stable cycling performance of the electrodes to the specific capacity expense. Therefore, we suggest that the SiO_2_ and/or void area may prevent the formation of rich Li_X_Si phases during lithiation and thus limit the cracking and pulverization of the nanoparticles during cycling. Controlling the void and SiO_2_ areas would greatly improve silicon-based anode technology.

## Figures and Tables

**Table 1:** Electrode Composition (% *w*/*w*).

Electrode	Si/SiO_2_	Graphene	Super P CB	CMC
Si	60%	-	20%	20%
Si_Gr	53%	6.7%	20%	20%
Si/SiO_2_	60%	-	20%	20%
Si/SiO_2__Gr	53%	6.7%	20%	20%
Si/void_Gr	53%	6.7%	20%	20%

**Table 2:** Comparison of electrochemical performance between our work and the reported work. For fair comparison, the capacity retention after 75 charging/discharging cycles, if possible, was considered. It has to be noted that the C rates for all references ranged from 0.1 to 0.5 A/g.

Reference No.	Cycle No	Capacity [mAh/g]	Capacity Retention [%]	Oxidation Method	Void Area [nm]
[41]	75	~1040	49.5	TEOS	10 & 50
[42]	75	~1149	49	TEOS	15, 20, 30, 40
[43]	75	~1599	56.6	TEOS	80–100
[44]	75	~698.5	50.4	Ambient Thermal	Ranging, <50 nm
[46]	75	~1735	73	TEOS	N.A. *
[47]	75	~867	45	MPTS	~100
[48]	20	~1291	57.7	Sol-gel reaction	~200
[49]	75	~1029	70.5	TEOS	80–100
Our Work	75	798	73	Oxygen Thermal	10–20
	75	872	63		

* N.A.: Not Available.

**Table 3:** Warburg coefficient values for all electrodes extracted from EIS measurements.

	Warburg Coefficient (Ω cm^2^)						
cycle	1	2	3	4	5	20	40
Si	0.390				0.254	3.91	
Si_Gr	0.057	0.052	0.042	0.057	0.058	0.057	0.062
Si/SiO_2_	2.433	3.022	7.473	10.316	10.317	10.6	10.8
Si/SiO_2__Gr	3.200	1.038	0.983	0.925	0.927	0.68	0.75
Si/void_Gr	0.114	0.911	0.918	1.086	1.101	2.16	2.6

## Data Availability

The data presented in this study are available on request from the corresponding author.

## Supplementary Materials

The following are available online at https://www.mdpi.com/article/10.3390/nano11112864/s1, Figure S1: Voltage vs gravimetric specific capacity of electrodes (a) Si, (b) Si_Gr, (c) Si/SiO_2_, (d) Si/SiO_2__Gr, and (e) Si/void_Gr, for the charge discharge cycles 1 to 5, 10, 20 and 40, in coin-type half-cell configuration.
