# Supplementary material for: Study of the Role of Void and Residual Silicon Dioxide on the Electrochemical Performance of Silicon Nanoparticles Encapsulated by Graphene
Source: Nanomaterials (Basel). 2021 Oct 27;11(11):2864. doi: 10.3390/nano11112864 (PMC8622490; doi:10.3390/nano11112864)
Supplement: Supplementary file 1 [file nanomaterials-11-02864-s001.zip › nanomaterials-1439296-supplementary.pdf]

nanomaterials

Supplementary Materials:

Study of the role of void and residual silicon dioxide on the electrochemical performance of silicon nanoparticles encapsulated by graphene.

D.-P. Argyropoulos [1,*], G. Zardalidis [1], P. Giotakos [2], M. Daletou [2] and F. Farmakis [1]

1 Electrical and Computer Engineering Dept., Democritus University of Thrace, Kimmeria Campus 67100 Xanthi, Greece; farmakis@ee.duth.gr
2 Foundation of Research and Technology, Hellas - Institute of Chemical Engineering Sciences, FORTH/ICEHT, Stadiou Str, Platani Rion, P.O. Box 1414, Patras GR-26504, Greece; riadal@iceht.forth.gr
* Correspondence: dimiargy56@gmail.com

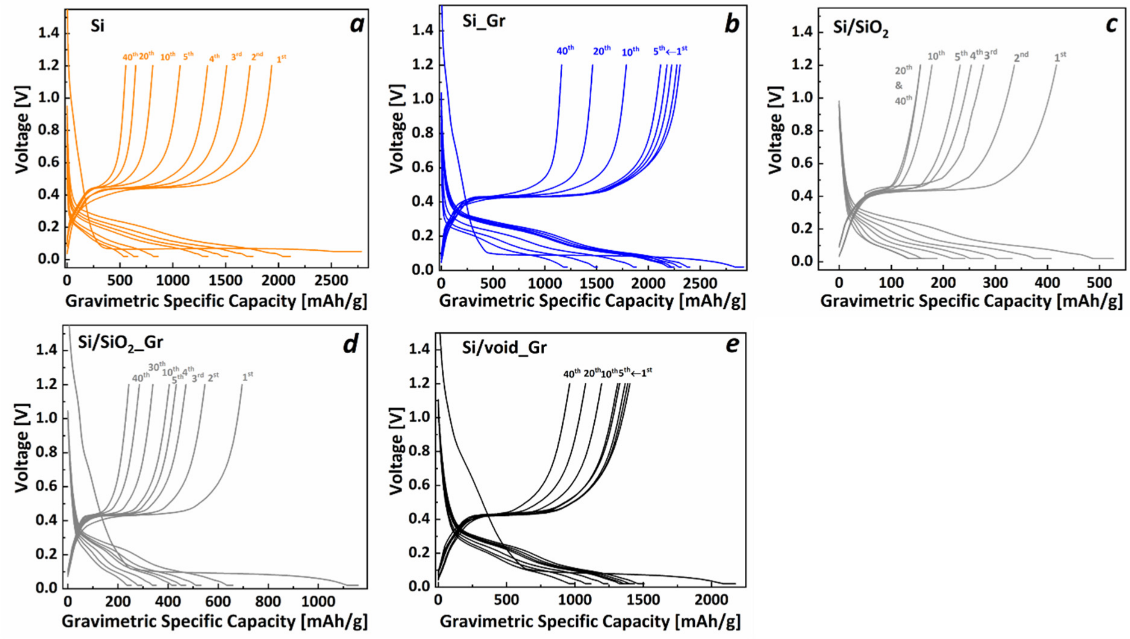

Figure S1: Voltage vs gravimetric specific capacity of electrodes (***a***) Si, (***b***) Si_Gr, (***c***) Si/SiO_2, (***d***) Si/SiO_2_Gr, and (***e***) Si/void_Gr, for the charge discharge cycles 1 to 5, 10, 20 and 40, in coin-type half-cell configuration.
